# A minimal conformational switching-dependent model for amyloid self-assembly

**DOI:** 10.1038/srep21103

**Published:** 2016-02-17

**Authors:** Srivastav Ranganathan, Dhiman Ghosh, Samir K Maji, Ranjith Padinhateeri

**Affiliations:** 1 Department of Biosciences and Bioengineering, IIT Bombay, Mumbai, India

## Abstract

Amyloid formation is associated with various pathophysiological conditions like Alzheimer’s and Parkinson’s diseases as well as many useful functions. The hallmark of amyloid assemblies is a conformational transition of the constituent proteins into a β - sheet rich filament. Accounting for this conformational transition in amyloidogenic proteins, we develop an analytically solvable model that can probe the dynamics of an ensemble of single filaments. Using the theory and Monte Carlo simulations, we show the presence of two kinetic regimes for the growth of a self-assembling filament – switching-dependent and –independent growth regimes. We observe a saturation in fibril elongation velocities at higher concentrations in the first regime, providing a novel explanation to the concentration-independence of growth velocities observed experimentally. We also compute the length fluctuation of the filaments to characterize aggregate heterogeneity. From the early velocities and length fluctuation, we propose a novel way of estimating the conformational switching rate. Our theory predicts a kinetic phase diagram that has three distinct phases – short oligomers/monomers, disordered aggregates and β -rich filaments. The model also predicts the force generation potential and the intermittent growth of amyloid fibrils evident from single molecular experiments. Our model could contribute significantly to the physical understanding of amyloid aggregation.

Protein aggregation is a phenomenon in which peptides/proteins self-associate to form oligomeric or higher-order structures that have wide implications in living systems. Amyloids are one such highly ordered protein/peptide aggregates that are typically associated with diseases like Alzheimer’s and Parkinson’s^1^. More recently, these structures have also been known to perform useful native functions in various biological hosts, ranging from the structural strength of spider silk^2^, to the storage of peptide hormones within secretory cells^3^. Owing to the crucial role that these assemblies play in both disease and functional contexts, it is vital to study these structures in greater detail.

Typically, during the process of amyloid aggregation, proteins/peptides undergo structural transition from their native conformations to a *β*-sheet rich state. The formation of the *β*-sheet can break the globular symmetry, seen in typical unstructured aggregates, and can give rise to linear assemblies. Structurally, amyloids can be defined as ordered protein fibres which are composed of *β*-strands that are perpendicular to the axis of the fibril to give rise to a typical structure known as the cross-*β*-sheet^4^. This conformational transition and cross-*β*-sheet rich nature is thus a hallmark of amyloid-like aggregates (also see Fig. 1A). The early stages of amylodogenesis is driven by the growth of this cross-*β*-sheet structure by self-recruitment of monomers along its linear axis. The growth of the cross-*β*-sheet could thus be considered to be the major step during amyloid formation.

Interestingly, the propensity to form these ordered structures is not restricted to any particular class of proteins but is exhibited by a diverse set of soluble proteins/peptides, under suitable experimental conditions^1^,^3^,^5^,^6^,^7^,^8^. A variety of experiments in bulk solution have shed light on the phenomenological similarities in amyloid formation like sigmoidal growth kinetics (fluorescence based methods, light scattering) and an increase in *β*-sheet content (circular dichroism (CD), fourier transform infrared spectroscopy (FTIR)) during self-assembly. Other experimental techniques like light scattering have been used to study the relationship between concentration and early growth velocities of a fibril^9^. More detailed single molecule studies have highlighted an intermittent, stop-and-go nature of the growth of individual filaments^10^,^11^.

Several theoretical models attempt to explain the properties of amyloid fibrils observed in bulk experiments, like sigmoidal growth kinetics. These models can be broadly classified as those based on primary growth processes^12^,^13^,^14^ and others which are based on secondary growth events^12^,^15^. Among the set of kinetic models that are based on primary growth mechanisms, the most prevalent are nucleation dependent polymerization (NDP) models. These models routinely include three major events—nucleation, polymerization and depolymerization^12^. However, such models do not explicitly account for the structural transition that is known to occur during fibrillation (increase in *β*-sheet content). Variations of this model employ size-dependent polymerization and depolymerization rates with distinct rates for aggregates that are smaller or larger than the size of a critical nucleus^16^,^17^. Such models have been successful in explaining the sigmoidal growth kinetics observed in case of amyloids along with other features including the effect of concentration on lag times as well as seeding^12^,^16^,^17^. Other kinetic models include those based on structural details of amyloid self-assembly wherein a rate-limiting monomer activation step corresponds to the partial misfolding or unfolding of a protein en route to aggregation^18^. In some cases, conformational changes at later stages of aggregation are considered where the aggregates could undergo reorganization into *β*-sheet like fibrils. Lomakin *et al.* described a framework of A*β* aggregation, wherein micelles of aggregating proteins were proposed to be sites of nucleation during the fibrillation process^9^. In this approach, the micelles are considered to be the source and sink of monomers, which cause a buffering effect on monomer concentration. The micelles are distinguishable from critical nuclei by not being aggregation competent.

Increased complexity in these kinetic models leads to difficulty in solving the equations mathematically. In the context of the current literature, we find that there exists a necessity to develop a generic model that takes into account crucial features like structural transition of proteins/peptides during aggregation while being simple enough to be mathematically solvable. Minimal models help to identify how the key primary events (polymerization, structural transition etc) influence the self-assembly process and their dynamics^19^,^20^. In the current study, we explicitly account for a key feature in amyloid aggregation, i.e structural transition from a coil like state to a *β*-sheet rich state which is commonly associated with diseases like Alzheimer’s and Parkinson’s. Using distinct polymerization and depolymerization rates based on the conformational state of the peptide, we make key observations related to amyloid-like self-assembly of single filaments. With an exactly solvable mathematical model, we explain phenomenon like concentration-dependence of growth velocities, fibril length diversity and intermittent nature of fibril growth which are not clearly understood previously.

## Model

Many of the existing computational approaches to understand amyloid aggregation have various limitations; (i) The prevalent method is to perform computationally intensive molecular dynamics simulations^21^,^22^,^23^,^24^,^25^,^26^. These simulations cannot typically access length scales beyond one or a few monomers and timescale beyond microseconds; these are much smaller than experimentally relevant protein aggregation length- and time-scales. (ii) Molecular simulations often cannot provide simple analytical insights into the problem and, (iii) are not very handy for an experimentalist who would want to explore different parameter regimes for various proteins and explore the phase space or fit with experimental data. To go beyond these limitations, we develop a coarse grained framework to describe the phenomenon of amyloid formation and protein aggregation in general.

### Definition of the model

**Figure 1:**
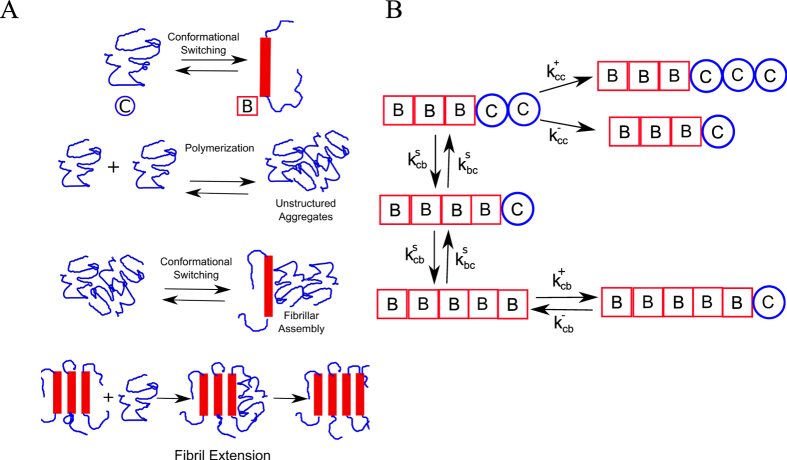
(**A**) A schematic depiction the phenomenon of amyloid-like self assembly by proteins/peptides. Initially, free monomers in solution are in their soluble form (state ‘C’). These monomers can stochastically self-associate to give rise to unstructured aggregates. Upon aggregating, they can undergo structural transition from a coil like state ‘C’ to a *β*-sheet like form (state ‘B’). These insoluble *β*-sheet rich assemblies are more stable due to specific H-bonded interactions and continue to grow by recruiting more monomers from solution. The initial state of the peptides in solution is its soluble form ‘C’. (**B**) The two-state model for amyloid formation showing the various processes implemented in this model. A polymerization event wherein a random-coil monomer ‘C’ binds to a random coil monomer at the fibril edge 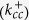, the switching of a C monomer to a B monomer on the filament 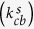, the switching of a B monomer to a C monomer on the filament 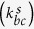 and the binding of a free C monomer to a B monomer on the fibril edge 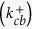 are shown in the figure. The depolymerization of a ‘C’ monomer bound to a ‘C’ monomer and a ‘C’ monomer bound to a ‘B’ monomer are 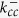 and 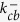, respectively. ‘C’ and ‘B’ refer to the coil-like and *β*-strand conformations of the peptide, respectively.

We assume that, at 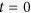, the monomeric protein/peptide in solution is either partially folded or unstructured having a coil-like configuration (see the blue units in Fig. 1 marked as “C”). This initial monomeric state “C” in our model could refer to a natively unstructured peptide/protein or an unstructured state of an otherwise folded protein. This monomer can undergo a conformational transition and switch to a different state which is partially ordered having a *β*-rich configuration (see red units in Fig. 1 marked “B”). However, the conversion from the coil-like state (C) to the *β*-like state (B) in solution is thermodynamically unfavorable (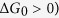 and there exists a large kinetic barrier^27^,^28^. We then assume that these C monomers can come together and weakly polymerise with an intrinsic rate constant 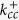. This C-C polymer is not thermodynamically stable and depolymerizes with a rate 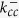 which is typically larger than 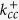 owing to the weak nature of the non-specific interaction within the C-C dimer^27^,^29^. Unlike in the solution, the C monomer on the filament can switch to the B state, with a rate 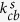, leading to a relatively more stable B-C complex. This B-C complex can further grow to a B-C-C state with a rate constant 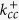 or disintegrate to a B state with a rate 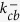. Since a solitary B monomer in solution is unstable, we assume that it instantaneously converts back to its unstructured state C. Another possibility is that the B-C state can switch to a B-B state which is highly stable compared to all other polymeric states and thus we consider the depolymerization rate 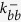 to be negligible. A ‘B’ monomer can switch back to its unstructured state C, at a rate 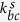 (see Fig. 1b); we assume that the switchings (forward and backward) occur in a sequential manner^30^. In other words, the switching of a monomer from the ‘C’ to ‘B’ state would be effected on the leftmost ‘C’ monomer. On the other hand, a reverse switching from state ‘B’ to ‘C’ would occur on the rightmost ‘B’ monomer.

### Master equations

If we consider an ensemble of single filaments having a set of reactions as mentioned above, their growth can be studied by writing down appropriate master equations and solving them to obtain experimentally measurable quantities such as growth velocities and fluctuations in lengths. Let 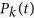 be the probability of finding *k* number of ‘C’ monomers at the tip of a growing filament at time *t*. 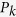 obeys the following master equation (for 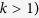:


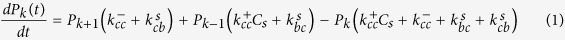


The first term represents events that lead to a decrease in the number of C monomers at the tip (depolymerization of C, and switching from C to B). The second term represents events that lead to an increase in the number of C monomers at the tip (polymerization of C, and switching from B to C). The third term is a combination of all events that will make the system leave its current state with respect to C monomers. 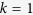 and 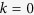 are special cases and one needs to write down modified forms of the above equations as shown in the Supplementary Text S1. In this paper, for mathematical simplicity, we assume that the backward switching rate 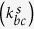 is negligible. The notation 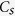 in our expressions refers to the constant free monomer concentration in solution. We can solve these set of equations at the steady state (at timescale much greater than all other timescales involved) by setting 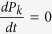 (see Supplementary Text S1); Note that the meaning of the steady state assumption here is that the distribution of the C monomers 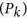 at the tip of the filament will become time-independent. It does not rule out computation of other time-dependent phenomena like B-dominated filament growth and time-dependent length fluctuations. While solving the equations, we find that there are two kinetic phases in the problem—one for 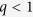 and another for 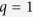 (see Results section for details), where, q is the probability of encountering at least one ‘C’ monomer at the tip of a growing filament, given by


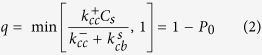


When 
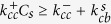
, the normalisation condition will demand that 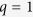 (see Supplementary Information Text S1 for details). Note that *q* can be experimentally controlled by varying the concentration 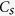. When 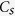 is less than a characteristic concentration 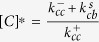, 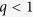. Conversely, when 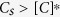, 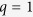.

For 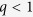, we can solve the master equations and obtain 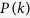 such that,


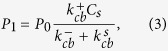



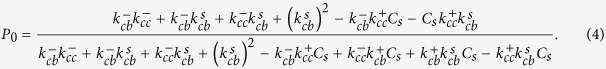


and, for 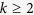,


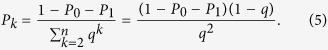


In some interesting limits, the expression for 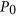 gets simplified. When 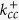 is much larger than other rates (to be precise, when 
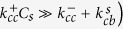
, 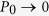. When the switching rate 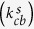 is much larger than all other rates, 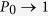. When 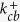 and 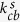 are both very large (compared to other rates), the system will tend to switch between two states defined by their 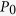 and 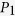 such that 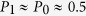. For any arbitrary rates, one can compute 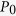 and 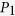 and immediately obtain experimentally measurable quantities such as growth velocity of the filament, and growth velocity of the *β* content, as we show in the results section.

### Simulations

We also performed kinetic Monte Carlo simulations^30^,^31^ using the events described in Fig. 1. The simulations were also used to compute measurable properties such as filament length, variance in fibril lengths, and to identify signatures of conformational transition-dependent growth. Our simulations were performed under two setups, i) conditions wherein the number of free monomers in solution is always maintained as a constant (we would refer to it as the “constant free monomer concentration” setup throughout the manuscript) and, ii) the condition wherein the free monomer concentration will gradually decrease upon polymerization. However, in this latter case, the total number of monomers in the system (free monomers plus the monomers on the filament) is conserved. We would refer to this as the “mass-conserved setup”. The two simulation setups are schematically depicted in the Supplementary Fig. S1. Additionally, simulations were also performed to show the applicability of the current analytical theory to predict growth velocities of aggregates, even in the presence of fibril breakage that leads to formation of new seeds. The setup for the fibril breakage simulations has been described in detail in the Supplementary Information Text S2.

## Results

### Two-state model: analytical solution, and the prediction of early filament growth velocities

Using the analytically solvable two-state model described above, we derive the expressions for growth velocities of protein aggregation and amyloid formation, assuming a constant free monomer concentration (under condition (i)). As mentioned in the Model section, two kinetic regimes emerge from the above model. In the first regime where there is a non-zero probability of encountering a completely *β*-rich filament 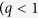 or equivalently 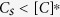, where 
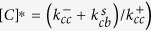
, the ensemble averaged growth velocity of a single filament can be obtained as:


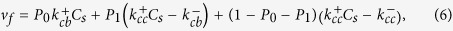


where 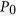 and 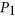 are the probabilities to find 0 and 1 ‘C’ monomer at the tip of the growing filament, respectively, as described in eq. 3 and 5. In the above expression for filament growth velocity, the first term represents the growth of the filament when it is completely in state *B*, and the last two terms represent the growth and shrinkage dynamics when there is at least one C monomer existing at the tip of the growing filament. Note that since 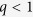 here, the steady state assumption 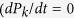, i.e the cap distribution is time-independent) is valid.

As the concentration exceeds the characteristic concentration [C]*, there is *always* one or more ‘C’ monomers at the tip of a growing filament. In this second regime 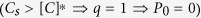, one can solve for the velocity expression without any steady-state assumption (see Supplementary Information Text S1):


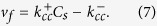


**Figure 2:**
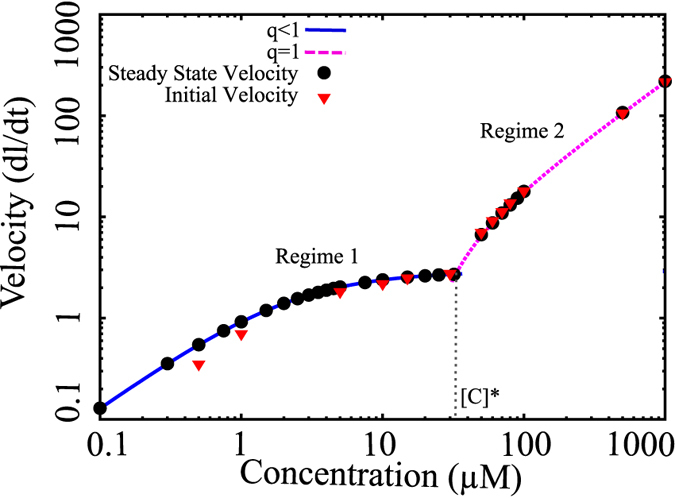
The concentration-velocity relationship shows two distinct regimes. The solid blue curve and the dotted pink curves are analytically computed growth velocities. The black filled circles and red filled triangles represent the steady state velocities (fixed concentration simulations) and initial growth velocity (mass-conserved simulations), respectively.

Since there are 1 or more C monomers always on the tip, the length change is essentially driven by addition of C monomers to a C monomer at the tip. In Fig. 2, we have plotted Eqs. 6 (solid curve, blue) and 7 (dashed curve, pink) as a function of free monomer concentration, [C].

Initially, the growth velocities show a concentration dependence before saturating at higher concentrations (solid curve, blue). However, beyond a certain concentration 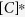, we observe a linear dependence of the growth velocities on concentration as given by eq. 7. Note that 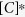 is the point where the blue and pink curves intersect. Above this concentration 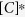, the aggregate grows by just polymerizing C monomers. To further validate our analytical calculations, we also performed kinetic Monte Carlo simulations using the same model (see Fig. 1), under constant concentration set up, and the results for the growth velocities are shown as black dots in Fig. 2. Similar to the prediction, the velocity of protein aggregation increases in a concentration-dependent manner, and shows two separate regimes. The prefect agreement between our theory and simulations validates the velocity formulae we derived.

Since typical experiments on amyloid fibril growth may not be performed under constant concentration set up as discussed above, we also performed simulations under mass-conserved conditions (condition (ii)). Under this condition, we compute the initial growth velocity of the filament. Initial growth velocity is defined as the average velocity in the growing phase until the filament grows up to 10% of its maximum length. These initial growth velocities are shown as red triangles in Fig. 2. Interestingly, the data shows that even under the monomer-depleting condition (mass conserved condition), the initial growth velocities also lie on two regimes similar to the steady state growth velocities computed analytically. This suggests that at large concentrations, for an initial period wherein the change in free monomer concentration is negligible, our velocity equations can compute early elongation velocities under the mass-conserved setup reliably.

From our analytical theory, we also calculate the velocity with which the *β*-sheet-content (the number of B monomers in our model) in the filament grows. In the first regime, 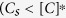 or 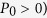 the *β*-growth velocity is given by:


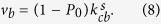


As we vary the parameter (in this case, increasing concentration of protein) to go from first regime to the second regime 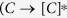 or 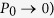, the *β* growth velocity becomes 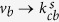. In the Supplementary Fig. S2, we have plotted 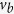 and compared with the overall filament growth velocity. This data suggests that for a particular sets of parameters, in the regime 1, filament velocity growth is comparable to the *β*-sheet growth velocity. This interesting fact suggests that regime 1 is essentially the regime relevant to amyloid growth in which the *β*-sheet growth velocity dictates the filament dynamics and must be studied in greater detail to understand the vital signatures of amyloid fibrillation.

### The saturation of the concentration-velocity curve indicates switching-dependent growth

**Figure 3:**
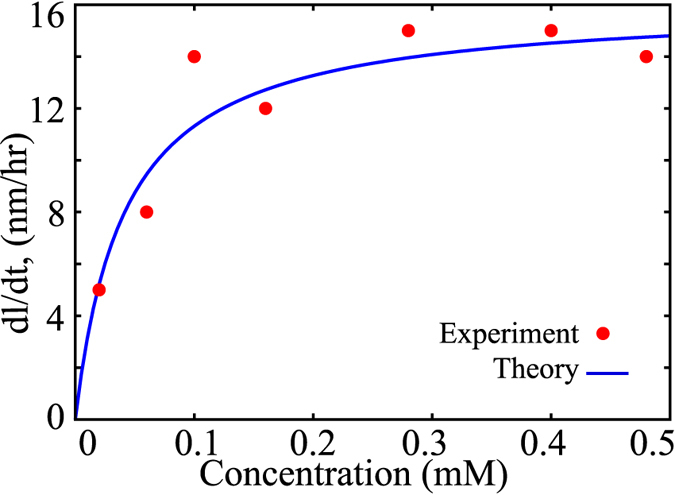
The analytical solutions for fibril growth velocity (solid blue curve) under constant concentration conditions shows a saturation at higher concentrations. This is in agreement to the experimentally reported saturation in growth velocities (red dots)^9^. In order to fit the experimental velocities to our theory, we used the following parameters; i) 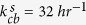, ii) The ratio of magnitude of rate constants, 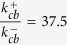 and values of other parameters such 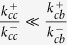 is satisfied (see text).

Based on our results which suggest that the first regime discussed above 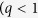; also see Fig. 2) is relevant to amyloid-like growth in experiments, we studied the concentration-velocity profile in this regime in detail as it will provide us a better understanding of the self-assembling system. A zoomed in version of the velocities in this regime is shown in Fig. 3 (blue, solid curve), which showed that the elongation velocities at large free monomer concentrations become essentially independent of concentration, leading to a saturation. Interestingly, when we analytically compute velocities at such large concentrations, we get


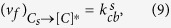


where 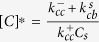 is the largest concentration possible in regime 1–the concentration beyond which regime 2 starts. The above result can be obtained by substituting 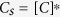 in eq. 7. At such high concentrations, the aggregate growth velocity is equal to the switching rate, 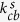, which is independent of concentration, resulting in the observed saturation. Moreover, at high concentrations, the *β*-sheet growth velocity is also equal to the switching rate, which is equal to the filament growth velocity. Therefore, in this regime, the filament grows by polymerization of C monomers immediately followed by conformational switching that stabilizes the filament. Interestingly, our results suggest that this saturation could be a unique hallmark of aggregates like amyloids that get stabilized after polymerization and promote further growth of the filament. Even though biofilaments like actin and microtubules are known to polymerize and undergo structural transformation (hydrolysis) on the filament, they do not display such a saturation as the monomer switching in those cases destabilizes the polymer.

A detailed understanding of this phenomenon can be obtained by analyzing each growth term in the velocity expression and is shown in Supplementary Fig. S4. The saturation seen here also provides us a novel way of estimating the switching rate experimentally; from eq. 9, it is clear that by measuring initial fibril elongation velocities as a function of protein concentration, we can obtain the switching rate directly. In fact, we found that similar saturation in growth velocities have been reported experimentally for amyloid-*β* protein fibril growth^9^. As shown in Fig. 3, we compare our predictions (solid curve) with one such set of experiments performed by Lomakin *et al.*^9^ (red dots) and obtain an approximate switching rate from the saturating velocities as 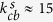 nm/hr ×2 monomers/nm = 30 monomers/hr. We multiplied with the factor 2 monomers/nm to account for the typical inter-*β*-strand-distance in a cross-*β* structure, which is 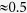 nm^4^. Our calculated initial velocities are comparable to the experimentally observed velocities for amyloid formation by A*β* peptide. As mentioned in the caption, even though many values that satisfy certain ratio of rates would fit the curve, the exact values used for the plot are: 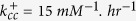, 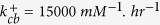, 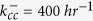, 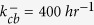, 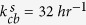. A more detailed rationale behind the fitting parameters is provided in Supplementary Text S2. In the Supplementary Fig. S3, we also present the effect of varying parameters on the concentration-velocity profile. We observe that increasing switching rate alters the overall nature of the first regime as well as the saturation velocities. On the other hand, varying 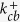 changes the concentration dependence at low concentrations, evident from the variation in the slope upon alteration in 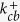. However, the polymerization rate 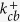 has no effect on the saturation point of the curve in the first regime (Supplementary Fig. S3).

### Estimation of force generation potential of a growing amyloid fibril

**Figure 4:**
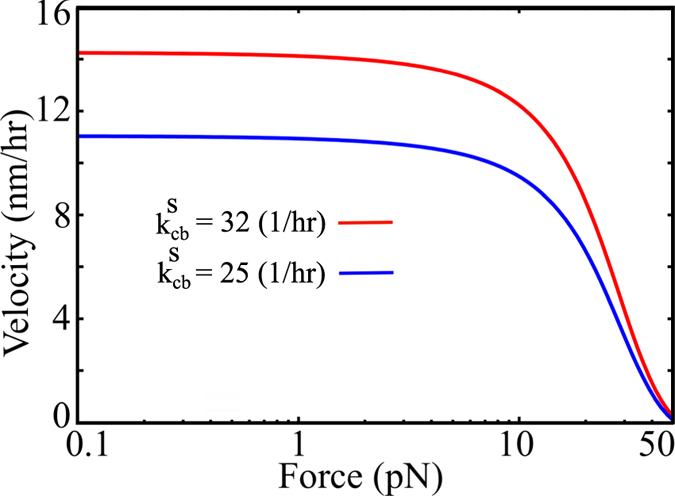
Theoretical estimation of force generation by a growing amyloid aggregate. The velocity is computed by imagining the filament growing against an opposing force which suppresses the polymerization rate as discussed in the text. The parameters used here are same as Fig. 3. The force-velocity profiles are plotted for a load distribution factor *α* = 1.

Polymerization of filaments have been well known to generate mechanical forces; typical examples being cytoskeletal filaments like actin^32^,^33^ and microtubule^34^,^35^. A recent study by Herling *et al*^36^ found that even growing insulin and lysozyme amyloid filaments have the ability to generate mechanical forces of the order of piconewtons per filament. However, there is no theoretical understanding of the underlying force-generation capacity of amyloid fibers. Using our linear aggregation model, we estimate the force generation potential of growing amyloid polymers under constant concentration conditions. To do this, we consider a setup wherein the filament is polymerizing against a “wall” (see Fig. S5 in Supplementary). The wall exerts a constant force such that the filament growth is suppressed, leading to rescaled polymerization and depolymerization rate constants 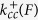, 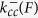, 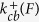 and 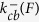, such that;


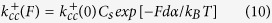



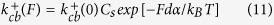



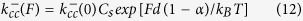



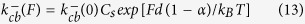


here 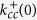, 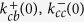, and 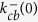 are rate constants in the absence of an external force while 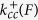, 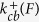, 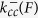, 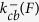 are the rate constants in response to an external load. *F* and *d* are the magnitude of the external force and the distance by which the wall would move (size of a monomeric unit) respectively. *α* is the load-distribution factor which dictates how the forces get distributed between the polymerization and depolymerization events. These modified rate constants were plugged into the velocity expressions given by equations 6 and 7 to obtain the force-velocity relation plotted in Fig. 4. For this force-velocity computation, we use the same kinetic parameters used in Fig. 3. As seen in Fig. 4, these parameters yield us forces of the order of piconewtons (per filament) which is comparable to the experimentally observed values for insulin and lysozyme fibrils^36^, thereby suggesting that the parameters are indeed sensible. Interestingly, we observe that in the regime of small forces, the velocities mainly remain unaltered (see Fig. 4). However, beyond a certain value of the external force, there is a steep decrease in growth velocities. This profile suggests that for small forces, any fluctuation in force of the order of ≈ pN will not alter the growth velocity significantly, suggesting that amyloid fibrils can act as stable agents to generate small amount of forces. Interestingly, this concave force-velocity profile that we predict for amyloid fibrils is in contrast to a convex profile observed in case of microtubules^37^. This difference in the force-velocity relationship could be another signature of amyloid fibril growth as a result of its stabilization due to conformational switching. In the Supplementary Fig. S6, we show the effect of varying the polymerization rate 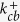 on the force-velocity relationship. The results suggest that for smaller 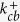 values, the velocities start declining at much lower external loads. The magnitude of external forces at which the velocities completely diminish are also much smaller. However, we see no such effect of varying switching rates on the values of stall force (Fig. 4). It must however be noted that the stall forces and the nature of the force-velocity profile could also vary depending on how the forces get distributed between the polymerization and depolymerization events as seen from the Supplementary Fig. S6B. Therefore, we believe that this simple model could serve as a guide for understanding the force generation mechanisms of growing amyloid fibrils and supplement future experiments. This estimation of force-velocity characteristics of amyloids may enable us to manipulate these structures as force generating systems in a more efficient manner, in addition to providing further insights into the mechanism by which they could act as membrane disrupting agents in disease.

### Kinetic phase diagram for protein aggregation

Protein/peptides during aggregation and amyloid formation access multiple states ranging from a functional monomeric state to disordered oligomeric form or an ordered amyloid-like self assembled form^38^,^39^,^40^. It is thus important to know the factors that govern the predominant state of a peptide/protein under any given conditions. To probe the multi-state behavior of self-assembling peptides, we systematically studied the effect of varying switching rates and concentrations on the filament and *β*-sheet growth velocities. To construct a phase diagram, we propose a critical growth velocity, *v*_*c*_ = 0.5 *μ*m/day; 0.5 *μm* is a length-scale that is close to the minimum length detectable using typical light microscopy.

**Figure 5:**
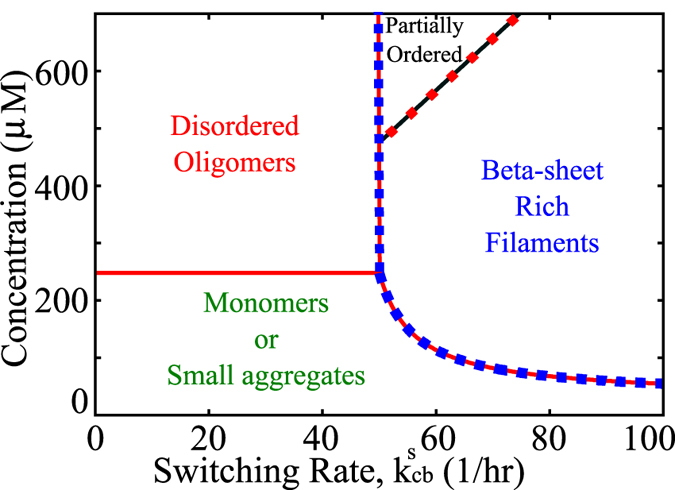
A kinetic phase diagram showing the effect of varying switching rate, 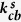 and free monomer concentration on the state of the protein/peptide. A peptide can remain monomeric or assume disordered, partially ordered or completely ordered aggregated states according to our model. We define the states as follows; i) 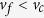 and 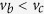, the peptide is in its monomeric form or exists as small oligomers. ii) 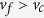 and 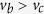 and 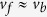, the amyloid-like ordered state dominates, iii) 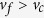 and 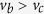 and 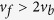, the aggregates are partially ordered with a mixture of *β*-sheet and unstructured content and iv) 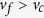 and 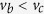, the aggregates are disordered in nature. 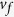, 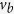 and 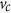 refer to the filament, *β*-sheet and critical growth velocities respectively.

In Fig. 5, the solid red curve is obtained by equating *v*_*f*_ = 0.5 *μ*m/day using eqs. 6 and 7. Similarly the dotted blue curve represents 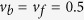 *μ*m/day. These curves can be defined as phase boundaries that demarcate different kinetic phases. At low concentrations and low switching rates, the growth velocity is too small (much below 0.5 *μ*m/day), and there will be negligible aggregation. Even at higher concentrations, if the switching rate is too small, amyloid-like *β*-rich assemblies will not be favored, rather the protein will form disordered/amorphous aggregates. For high switching rates, the velocity curve becomes non-linear; as switching rate increases, we observe more *β*-rich aggregates. In this high 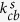 regime, we find 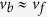 suggesting that the whole filament growth is essentially driven by *β*-sheet formation. However, for a given switching rate, there is a critical concentration that is required for amyloid growth.

Interestingly, at super-high concentrations and high switching rates, one would encounter a regime where both the *β*-sheet content and the filament grow faster than the critical growth velocity, 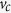, without being equal. The solid black line (Fig. 5) represents the critical condition wherein the net polymer aggregation velocity is more than twice that of the *β*-content growth, implying that the filament would be composed of mostly disordered structures with a partial ordered nature owing to the monomers that have already undergone conformational transition to the B state. We define this state, which is neither completely ordered nor entirely disordered as ‘partially ordered’ aggregates. The present calculation thus provides a set of mathematical expressions for deriving the growth velocities/critical concentrations/phase boundaries for protein aggregation and amyloid formation without any computer simulation.

### Species diversity is regulated by conformational switching and is maximum during the phases of highest growth

So far we have been discussing average (mean) quantities like growth velocity, under constant concentration conditions. We further investigate higher order statistical quantities like variances that can provide us an insight into the diversity of filament populations at various times during the growth. This is extremely relevant given that recent studies have reported a wide distribution of filament sizes and a potentially toxic nature of oligomers, as opposed to stable long filaments^41^,^42^,^43^,^44^.

**Figure 6:**
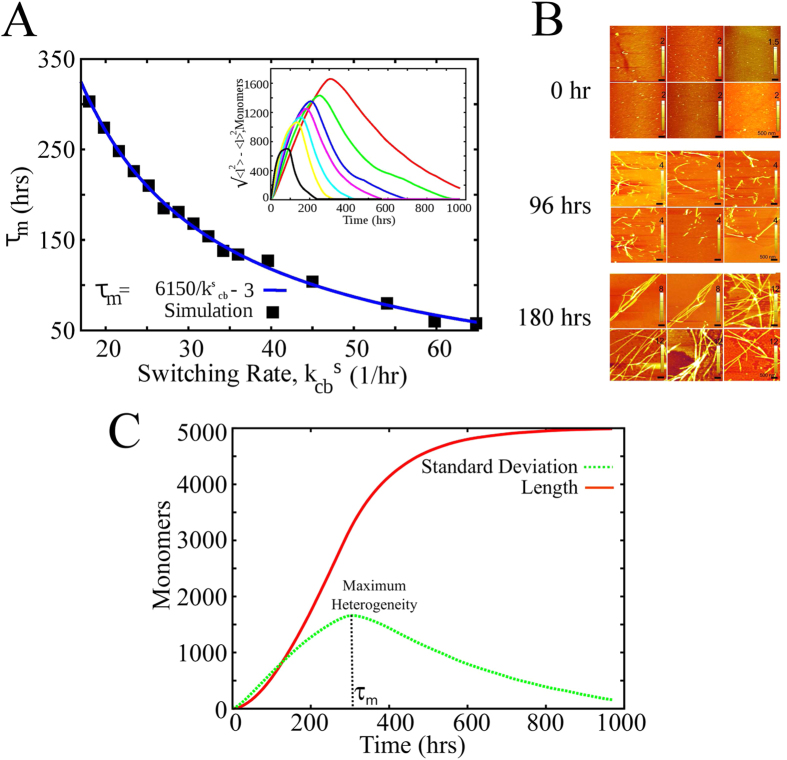
(**A**) Relationship between the time of observation of maximum divergence, 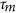 and the conformational switching rate, 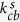. Inset shows filament length heterogeneity at various times during fibril growth. Each curve in the inset represents a standard deviation versus time plot for different values of conformational switching rate 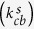. (**B**) Atomic force micrographs at various time-points during *α*-synuclein fibrillation kinetics showing a homogenous initial population with negligible aggregation. At an intermediate timepoint, small aggregates coexist with longer filaments, thereby indicating a higher heterogeneity. At later timepoint, most of the filaments mature into longer fibrils. The scale bar in the AFM images is 500 nm. Height scales are depicted in individual AFM images. (**C**) A representative figure showing the filament length heterogeneity (*σ*) and the average length as a function of time from our mass-conserved simulation. The definition of 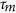 is also shown in the figure.

To get insight into the time-dependent nature of diversity, we computed the standard deviation in fibril lengths as


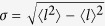


at various time points during the Monte Carlo simulations. Note that the angular brackets ‘
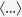
’ denote the averages over various realizations (ensemble average). We have done this both for constant concentration (Supplementary Fig. S7) and monomer-depleting conditions (mass-conserved setup). Given that the latter condition is more common in experiments, we have presented this result in Fig. 6A (inset). Our findings suggest that the variance in fibril lengths continues to increase during the growth phase, peaks and then reduces as more and more filaments reach their equilibrium lengths. This may also be observed in experiments. As an indicator, we have shown AFM images during various time-points of *α*-synuclein (associated with Parkinson’s disease) fibrillation kinetics (Fig. 6B). The micrographs suggest negligible aggregation at an early timepoint resulting in greater homogeneity. However, at an intermediate timepoint, smaller aggregates coexist with longer filaments, thereby indicating a higher heterogeneity. At later timepoint, most of the filaments mature into longer fibrils. These results suggest a non-monotonicity in fibril length heterogeneity. However, owing to limitations such as the possibility of mature filaments at later timepoints spanning beyond the AFM field, we do not provide a quantification of the variance in lengths. Another measure of heterogeneity 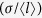 as a function of time is shown in Supplementary Fig. S8B which shows a gradual decrease with time suggesting that uniformly longer filaments dominate at longer timescales.

A previous study by Arosio *et al.*^45^ monitors the time evolution of fibril length distributions for *β*-lactoglobulin (*β*-LAC). Their results suggest a gradual increase in length heterogeneity from 5 hrs to 8 hrs followed by a decrease in length heterogeneity around 48 hrs under stagnant conditions, similar to our prediction. However, under shaking conditions such a behavior is not observed which could be due to fragmentation of filaments at 600 rpm. In this study, we attempt to identify the characteristic profiles of the temporal evolution in length heterogeneity that can emerge from a model devoid of secondary processes like fibril breakage. Furthermore, even in a scenario where the breakage length for the fibrils is long or if the breaking occurs at later timescales during the kinetics, the predictions of the current model would still be valid. Interestingly, we do find reports of such instances (in insulin and *β*-lactoglobulin) wherein fibril breakage occurs only at later timepoints of the growth when most of the free monomers have been consumed^46^.

The natural question to ask is what determines the timescale at which the heterogeneity is maximum? When we investigated the role of different parameters on heterogeneity, we found that conformational switching shifts the timescale 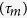 over which the variance reaches its maximum (see Fig. 6A, inset and Fig. 6C), in addition to modulating the extent of heterogeneity. When we plotted the timescale 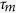 against the switching rate (Fig. 6A), we found an inverse relationship where 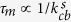. This data suggest that for high switching rates the peak of the variance would appear earlier compared to low switching rate. Further this may also provide the conformational switching rate from experimentally observed filament length heterogeneity.

### Stop-and-go growth in amyloid fibrils emerges from the model

**Figure 7:**
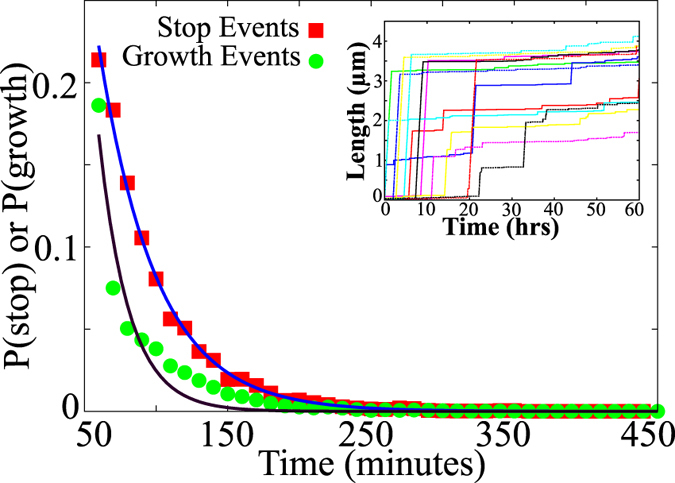
Distribution of growth timescales (green, circles) and stop timescales (red, squares) computed over the first 60 hours of filament growth. The curves are exponential functions plotted as a guide to the eye. The inset shows filament growth kinetics from our model exhibiting stop and go kinetics. The results are consistent withe experimental findings^10^,^11^. The parameters used here are 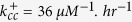, 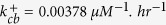, 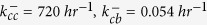, 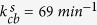.

Typical measurements of fibrillar growth involve fluorescence based measurements which reflect the extent of fibrillation in the bulk solution. However, with the availability of techniques which allow amyloid fibrillation to be probed at a single filament level^47^,^48^, more interesting features of filament growth have been identified. Recent studies reveal that many proteins including *α*-synuclein, *β*2-microglobulin have shown a stop and grow kinetics during their fibril growth, instead of continuous growth of the fibrils^10^,^11^. Earlier models^10^,^11^ were specifically devised to capture this behavior of individual filaments by introducing extra “stop” and “start” states, by hand, which did not have any structural support from the known literature. Here, we find that the stop-and-go nature of fibril growth naturally emerges from our model–a model based on the well known phenomena of secondary structural transitions. To test whether our model can predict this “stop and go” kinetics of fibril growth, we plot length vs time for a new set of parameters. In Fig. 7 (inset), we plot length as a function of time for various realizations of the filaments with parameter values being 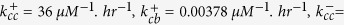

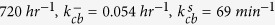
.

As reported by the experimental studies on single filament growth^10^,^11^, the distributions of the start and stop timescales also show a great degree of similarity (see Fig. 7), indicating that both start and stop phases are equally probable during filament growth. Furthermore, both the duration of the start and stop events follow an exponential distribution, similar to the previous experimental findings^10^,^11^. We found that this intermittent growth of the individual fibrils emerges towards the end of the first regime (*q* very close to 1), wherein one of the polymerization rates is significantly lower than the other, leading to the aforementioned pause in growth. We have defined the stop event as a phase during which the growth rate is less than 2 nm/min, consistent with the experiments. With the emergence of powerful single molecular analysis tools, our model could thus have wide applicability in understanding the dynamics of single-filament growth.

## Discussion

### The need for a generic model

Understanding the mechanism of protein aggregation and the role played by various factors in modulating the nature and the dynamics of aggregation is of vital importance. Given the broad similarities in the characteristic profile of amyloid-assemblies irrespective of their sequence and biological function, it is important to formulate an effective generic model for amyloid formation. Multitude of environmental factors, viz. pH, ionic strength, presence of co-aggregating proteins and/or inducer molecules have been previously reported to play a significant role in promoting protein aggregation^8^,^49^,^50^,^51^,^52^,^53^,^54^. These factors can not only modulate the kinetics of proteins with predisposition to aggregate, but also completely change the aggregation dynamics of usually non-aggregating proteins^8^,^50^,^52^. The precise mechanisms by which these factors alter the aggregation dynamics is unclear. Any alterations at the sequence level or in solution conditions could have potential manifestations at more macroscopic lengthscales and timescales. While molecular simulations may give us insights into the protein interactions at a microscopic level, it is important to formulate a coarse-grained framework that provides us with a more macroscopic understanding of the self-assembly process.

### Analytical solvability and structural features

In this study, we devise a model that is mathematically tractable, computationally less demanding and inclusive of phenomenological details of protein aggregation. The key feature of our model is the incorporation of a conformational switching rate that governs fibril growth and extension—the typically-observed secondary structural transition from an initial state to a *β*-sheet rich state^55^. Our linear two-state model comprising of polymerization, depolymerization and switching events is analytically solvable under conditions of constant free monomer concentration. Owing to its mathematical tractability, it could be a powerful tool that not only provides us with a mechanistic understanding of amyloid aggregation but could also be a useful guide while designing amyloid-aggregation based experiments. Additionally, our results also provide a method to obtain the hitherto unknown model parameters like the rate of conformational transition during aggregation. This study sheds light on the minimal set of parameters that could be essential to model the phenomenon of protein aggregation. Our work demonstrates the efficacy of models devoid of microscopic details in understanding complex self-assembling systems.

### Novel signatures of amyloid aggregation

Typical signatures of amyloid growth include features like *β*-sheet propagation and sigmoidal growth kinetics. While these features provide us a method to monitor amyloid fibrillation, it would be useful to identify other kinetic signatures of amyloidogenesis. One of the key findings of our model is the presence of two kinetic regimes for amyloid fibril growth, each with a distinct dependence on concentration. The first regime (Fig. 2, blue solid line) which shows an initial monotonic increase in growth velocities followed by a saturation in velocities is a potential signature of conformational-transition dependent growth. However, in the second regime above a critical concentration 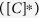, we predict a linear dependence on the free monomer concentration (Fig. 2, pink dotted line). Interestingly, the magnitude of the saturation velocity in the first regime is limited by the the switching rate itself.

Our results suggest that this saturation of growth velocities at higher concentrations in the first regime could be a signature of polymerizing systems that are accompanied by conformational switching. In the absence of the stabilization provided by the conformational transition, further extension would be disfavored due to very weak polymerizing tendency. Therefore, such filaments grow by polymerization that is accompanied by conformational switching that leads to disfavored depolymerization, allowing them to elongate further. Crucially, such a saturation in elongation velocities that emerges from our model has also been reported experimentally^9^. Our framework provides a novel structural and conformational basis for this experimentally observed saturation. We therefore suggest that the concentration-velocity profile could be used to characterize the nature of aggregation, with saturation of growth velocities at higher concentrations pointing towards self-assembly that is accompanied by conformational transition which stabilizes the aggregate.

The analytically computed growth velocities also conform to the early stage growth velocities computed from our mass-conserved simulations. This indicates that even in traditional experimental setups where there is depletion in free monomer concentration during polymerization, our model can be used to predict the early growth kinetics reliably. The kinetic Monte carlo simulations performed under a mass-conserved setup point towards another interesting signature of amyloid aggregation which is the relationship between the rate of conformational transition and diversity in filament lengths. The time-dependence of the heterogeneity reveals interesting aspects of conformational-transition dependent aggregation. The filament length heterogeneity continues to increase till a certain time during the growth, declines and eventually stabilizes owing to the stable nature of *β*-rich fibrils (Fig. 6). The time at which this peak (Fig. 6) in variance is observed showed an inverse proportionality to the conformational switching rate, 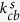. Thus, our model predicts that the time taken to observe the maximum heterogeneity in filament length could be another vital indicator of the amyloidogenic propensity of any protein/peptide. Additionally, the extent of diversity also decreases with an increase in the rate of conformational switching, suggesting that any change in environmental conditions that promote *β*-sheet development or stabilizes the steric zipper structure could alter the heterogeneity of system. We predict that a greater propensity to access the *β*-rich state would result in lower diversity in filament lengths. This might have implications on the potential toxic nature of these aggregates, with evidences pointing towards the role of smaller filaments in causing cell damage^12^,^38^.

### Amyloid fibrils could act as stable agents against external forces in the order of piconewtons

Based upon the expressions that we derived for filament growth velocities, we also aimed at estimating the extent of force that these filaments could potentially generate. Although, there is no documented literature of amyloid-like fibrils being exploited for their force-generation ability *in vivo*, an understanding of their force generation potential could be of vital interest while exploiting the material properties of amyloids. Recent *in vitro* studies also reveal the force generation potential of these fibrils^36^. Using our model, we attempt to provide a generic framework for the plausible force generation potential of amyloids. The force-velocity profiles predicted in the current study could also serve as a guide for understanding the growth of these structures in greater detail. For instance, the concave force-velocity curve that we predict for amyloid fibrils is contrary to the convex profile observed in case of cytoskeletal filaments like microtubules. This could emerge from the stabilizing nature of structural transition within amyloids. Additionally, our study could supplement future experiments and help us understand how the forces get distributed between polymerization and depolymerization events.

Our study further reiterates the ability of growing amyloids to generate forces in the order of piconewtons, comparable in magnitude to the forces generated by native mechanical force generators of the cell like the cytosekeletal filaments. This might have potential ramifications in situations where polymerization is aberrant, unregulated and detrimental to the cell, like in neurodegenerative diseases like AD and PD which are known to be major causes of cell death and tissue damage. Our theoretical prediction of the force-velocity relationship for these fibrils reveals that these filaments could be extremely resistant to small fluctuations in forces in the order of piconewtons. Therefore, a large buildup of such aberrantly polymerized structures could lead to forces, potentially large enough to cause cell and tissue damage.

### Multi-phasic nature of proteins/peptides

A plethora of studies have shown that a wide range of proteins could be driven to access the amyloid-state upon modulating various environmental conditions^8^,^49^,^50^,^51^,^52^,^53^,^54^. This suggests that the same protein/peptide could remain in its functional form, assume a disordered aggregated structure or assemble into amyloid-like structures based upon the solution conditions. Using our analytical expressions for growth velocities of the aggregate 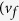 and 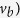 in equations 6 and 7, we construct a phase diagram which can similarly predict multiple states of the peptide with monomer concentration and switching rate 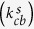 being our phase parameters. The phases predicted from our model are based on the assumption of an arbitrary critical growth velocity with which the filament has to grow in order for visible aggregation to occur at meaningful timescales. The various phases are based on the overall aggregate velocity as well as *β*-sheet growth velocities reaching this critical velocity at any point on the phase space. It must be noted that while our choice of critical growth velocity used to construct the phase diagram in Fig. 5 might be arbitrary, the same could be extended to any value of critical velocity which might be experimentally meaningful. Our findings suggest three major phases which the peptide/protein could access depending upon the two parameters. Below a certain critical concentration, the filament growth velocities would be negligibly low and there may not be any visible signs of protein aggregation at biologically relevant timescales, enabling the scavenging and clearance mechanisms within the cell to act on these incorrectly aggregated structures. Even upon reaching this critical concentration, the protein/peptide can exist in any of its two self-assembled states based upon a second parameter, which in this case is the switching rate. A similar observation for the non-linear dependence of amyloid-growth on concentration has also been previously reported by Schmit *et al.*^56^ wherein they report that any change in solution conditions which might stabilize the formation of a steric zipper could lead to a shift in equilibrium from the disordered oligomeric state to the fibrillar state. From our model, we speculate that the conformational switching rate 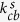 could thus not only be an intrinsic property of the peptide which might characterize its amyloidogenic propensity but also a factor that can be modulated by other external factors like pH, salt concentration, ionic strength of the buffer or the presence of co-factors that promote amyloidogenesis. This is supported by experiments which suggest that presence of glycosaminoglycans, co-factors or ions could enable a change in morphology of a protein/peptide^39^,^49^,^51^,^52^,^53^. Thus, by modulating the environmental factors or even due to sequence level effects, the predominant phase could could be shifted from one phase to the other.

Contrary to early studies which regarded amyloid fibrils to be the causative agents for neurodegenerative diseases, more recent studies have suggested that stabilizing the aggregates in their stable amyloid-like state could actually lead to a reduction in toxicity^38^,^39^,^41^. These suggestions are in line with studies which suggest that disordered oligomeric species could result in cell death owing to their higher hydrophobic surface exposure^57^,^58^. It is thereby imperative to understand the factors that govern the predominant state in which a peptide exists in response to varying environmental and intrinsic factors. Our theoretical framework, in principle demonstrates the ability to account for this multi-state behavior of peptides/proteins. A knowledge of such a phase space for a protein/peptide could thus help us significantly while devising strategies to tackle protein aggregation or while trying to enhance the self-assembling properties of a peptide/protein.

### Applicability of the constant concentration analytical theory for understanding amyloidogenesis

**Figure 8:**
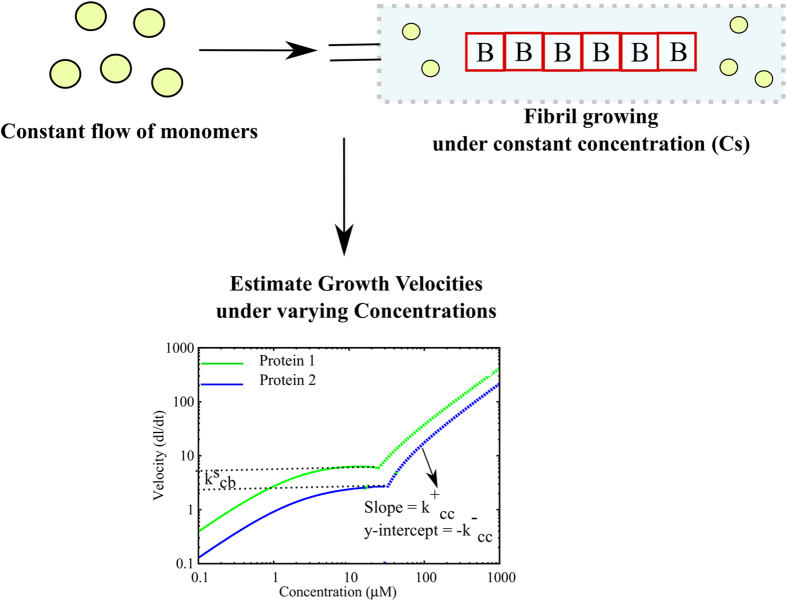
Suggested experimental design using the analytical theory. Experiments could be performed under conditions of constant free monomer concentration by constantly flowing in free monomers through a flow setup. The filament growth velocities under varying concentrations could be used to compute the growth parameters for different proteins. The framework could thus be used to compare amyloidogenic propensities of different proteins.

Our linear aggregation theory and the analytical expressions have been developed under the assumption of constant free monomer concentration. Traditional *in vitro* experiments are performed under depleting mass conserved conditions wherein the monomer concentration progressively gets depleted. However, experiments can be performed wherein monomers are constantly flowed in to maintain a constant concentration of monomers in the solution^36^. Experiments performed under such a carefully controlled fixed concentration setup could be used in conjunction with the current theory to enable us to gain insights into the intrinsic amyloidogenic tendencies of various proteins (Fig. 8). For instance, the current model, along with single filament growth experiments performed under a constant concentration setup could allow one to understand the mechanism by which a mutation could alter amyloidogenic property of a protein. This can be achieved by identifying the rates (in the current model) that get altered upon introduction of a mutation. This is possible due to the fact that growth velocities of any filament under the fixed concentration assumption would be unaffected by secondary processes like fragmentation. This model could thus act as a generic framework upon which we can compare the differences in amyloidogenic tendencies of various peptides.

Traditionally, amyloid aggregation has been modeled using primary growth events like nucleation, polymerization and depolymerization. However, recent studies have shown the role of secondary events like fibril fragmentation in the aggregation kinetics of amyloids^15^,^59^,^60^. Our model does not include any contribution of secondary growth events in the growth kinetics. The objective of the current work is to understand the growth of a seed in solution given a free monomer concentration. The analytical solutions for the growth velocities represent the rate with which any seed (both primary and secondary seeds formed due to processes like fragmentation) would grow under a given condition. Therefore under conditions of fixed free monomer concentration, even a secondary nucleus which forms upon fragmentation would grow at velocities predicted by our analytical solutions. In order to further emphasize on this point, we performed simulations under the constant concentration setup, in the presence of fibril breakage. The details of the setup is described in Supplementary Text S2 and the model is schematically depicted in Supplementary Fig. S9A. In Fig. S9B, we show the lengths for various growing fragments as a function of time, using an extension of our model in which a growing filament can also fragment at some rate, under constant free monomer concentration, leading to formation of new seeds for growth. The results show that each new seed that results after fragmentation grows with similar steady state velocities, as apparent from their identical slopes. Experiments performed under conditions where there is a constant supply of free monomers in solution, under the current kinetic framework could thus yield us insights into the native tendencies of proteins/peptides to aggregate into *β*-sheet rich structures. Although typical experimental studies are performed under conditions of depleting monomer concentration, the current model may still be used to predict the early growth velocities for every new seed (under monomer concentration at that instant) formed upon fragmentation. However, the current model might not be suited for comparison with bulk experimental studies.

### Connecting the theory with experiments

One of the important applications of this model is its potential to be used as a tool for designing experiments that could help us understand the phenomenon of amyloidogenesis in greater detail. As discussed earlier, the concentration-velocity relationship could yield vital kinetic information about the polymerizing protein/peptide. Since velocities are measurable experimentally, in principle, we can get many of the amyloid fibril kinetic parameters by comparing our theoretical predictions with experimental data, if available. For example, if one can measure velocity in regime 2, by fitting a straight line (eq. 7), one should obtain values for 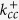 and 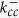. Similarly, the saturation point of the concentration-velocity profile would yield us the conformational transition rate, 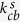. These rates could be useful in defining the propensity of various peptides/proteins to form amyloid-like structures. Additionally, these parameters could yield further insights into the mechanism by which various agents like mutations and solution conditions could modulate the fibrillation kinetics of amyloids by probing the rates that get altered. Furthermore, with the knowledge of the relationship between conformational switching rate and filament length heterogeneity, experiments could be setup to study the diversity in filament lengths under varying conditions favoring or disfavoring conformational transition. This would help us understand the propensity of the protein/peptide to undergo a transition to the *β*-rich state and the mechanism by which aggregation inducers might promote amyloid formation.

## Additional Information

**How to cite this article**: Ranganathan, S. *et al.* A minimal conformational switching-dependent model for amyloid self-assembly. *Sci. Rep.* **6**, 21103; doi: 10.1038/srep21103 (2016).

## Supplementary Material

Supplementary Information

## References

[b1] Chiti F. & Dobson C. M. Protein misfolding, functional amyloid, and human disease. Annu. Rev. Biochem. 75, 333–366 (2006). 16756495 10.1146/annurev.biochem.75.101304.123901

[b2] Kenney J. M., Knight D., Wise M. J. & Vollrath F. Amyloidogenic nature of spider silk. Eur. J. Biochem. 269, 4159–4163 (2002). 12180993 10.1046/j.1432-1033.2002.03112.x

[b3] Maji S. K. *et al.* Functional amyloids as natural storage of peptide hormones in pituitary secretory granules. Science 325, 328–32 (2009). 19541956 10.1126/science.1173155 PMC2865899

[b4] Greenwald J. & Riek R. Biology of amyloid: structure, function, and regulation. Structure 18, 1244–60 (2010). 20947013 10.1016/j.str.2010.08.009

[b5] Villegas V. *et al.* Protein engineering as a strategy to avoid formation of amyloid fibrils. Protein Sci. 9, 1700–1708 (2000). 11045616 10.1110/ps.9.9.1700 PMC2144697

[b6] Litvinovich S. V. *et al.* Formation of amyloid-like fibrils by self-association of a partially unfolded fibronectin type III module. J. Mol. Biol. 280, 245–258 (1998). 9654449 10.1006/jmbi.1998.1863

[b7] Obson C. H. M. D. *et al.* Amyloid fibril formation by an SH3 domain. Proc. Natl. Acad. Sci. USA 95, 4224–8 (1998). 9539718 10.1073/pnas.95.8.4224 PMC22470

[b8] Chiti F. *et al.* Designing conditions for *in vitro* formation of amyloid protofilaments and fibrils. Proc. Natl. Acad. Sci. USA 96, 3590–3594 (1999). 10097081 10.1073/pnas.96.7.3590 PMC22338

[b9] Lomakin A., Chung D. S., Benedek G. B., Kirschner D. A. & Teplow D. B. On the nucleation and growth of amyloid b-protein fibrils: Detection of nuclei and quantitation of rate constants. Proc. Natl. Acad. Sci. USA 93, 1125–1129 (1996). 8577726 10.1073/pnas.93.3.1125 PMC40042

[b10] Ferkinghoff-Borg J. *et al.* Stop-and-go kinetics in amyloid fibrillation. Phys. Rev. E 82, 7–10 (2010). 10.1103/PhysRevE.82.010901 20866557

[b11] Wördehoff M. M. *et al.* Single Fibril Growth Kinetics of *α*-Synuclein. J. Mol. Biol. 427, 1428–1435 (2015). 25659910 10.1016/j.jmb.2015.01.020

[b12] Gillam J. E. & MacPhee C. E. Modelling amyloid fibril formation kinetics: mechanisms of nucleation and growth. J. Phys.: Condens. Matter 25, 373101 (2013). 23941964 10.1088/0953-8984/25/37/373101

[b13] Harper J. D. & Lansbury P. T. Models of amyloid seeding in alzheimer’s disease and scrapie:mechanistic truths and physiological consequences of the time-dependent solubility of amyloid proteins. Annu. Rev. Biochem. 66, 385–407 (1997). 9242912 10.1146/annurev.biochem.66.1.385

[b14] Powers E. T. & Powers D. L. The Kinetics of Nucleated Polymerizations at High Concentrations: Amyloid Fibril Formation Near and Above the “Supercritical Concentration”. Biophys. J. 91, 122–132 (2006). 16603497 10.1529/biophysj.105.073767 PMC1479066

[b15] Knowles T. P. J. *et al.* An analytical solution to the kinetics of breakable filament assembly. Science 326, 1533–1537 (2009). 20007899 10.1126/science.1178250

[b16] Hall D. & Edskes H. Silent prions lying in wait: a two-hit model of prion/amyloid formation and infection. J. Mol. Biol. 336, 775–86 (2004). 15095987 10.1016/j.jmb.2003.12.004

[b17] Cohen S. I. A., Vendruscolo M., Dobson C. M. & Knowles T. P. J. Nucleated Polymerisation in the Presence of Pre-Formed Seed Filaments. Int. J. Mol. Sci. 12, 5844–5852 (2011). 22016630 10.3390/ijms12095844 PMC3189754

[b18] Roberts C. J. Non-Native Protein Aggregation Kinetics. Biotechnol. Bioeng. 98, 927–938 (2007). 17705294 10.1002/bit.21627

[b19] Morris A. M., Watzky M. a., Agar J. N. & Finke R. G. Fitting neurological protein aggregation kinetic data via a 2-step, minimal/“Ockham’s razor” model: the Finke-Watzky mechanism of nucleation followed by autocatalytic surface growth. Biochemistry 47, 2413–27 (2008). 18247636 10.1021/bi701899y

[b20] Watzky M. a., Morris A. M., Ross E. D. & Finke R. G. Fitting yeast and mammalian prion aggregation kinetic data with the Finke-Watzky two-step model of nucleation and autocatalytic growth. Biochemistry 47, 10790–800 (2008). 18785757 10.1021/bi800726m

[b21] Klimov D. K. & Thirumalai D. Dissecting the assembly of A β16-22 amyloid peptides into antiparallel beta sheets. Structure 11, 295–307 (2003). 12623017 10.1016/s0969-2126(03)00031-5

[b22] Cecchini M., Rao F., Seeber M. & Caflisch A. Replica exchange molecular dynamics simulations of amyloid peptide aggregation. J. Chem. Phys. 121, 10748–10756 (2004). 15549960 10.1063/1.1809588

[b23] Wang J., Tan C., Chen H.-F. & Luo R. All-atom computer simulations of amyloid fibrils disaggregation. Biophys. J. 95, 5037–5047 (2008). 18757563 10.1529/biophysj.108.131672 PMC2586582

[b24] Cecchini M., Curcio R., Pappalardo M., Melki R. & Caflisch A. A molecular dynamics approach to the structural characterization of amyloid aggregation. J. Mol. Biol. 357, 1306–21 (2006). 16483608 10.1016/j.jmb.2006.01.009

[b25] Ma B. & Nussinov R. Molecular dynamics simulations of alanine rich *β*-sheet oligomers: Insight into amyloid formation. Protein Sci. 11, 2335–2350 (2002). 12237456 10.1110/ps.4270102 PMC2373704

[b26] Miller Y., Ma B. & Nussinov R. Polymorphism in Self-Assembly of Peptide-Based *β*-Hairpin Contributes to Network Morphology and Hydrogel Mechanical Rigidity. J. Phys. Chem. B. 119, 482–490 (2015). 25545881 10.1021/jp511485n PMC4298354

[b27] Šarić A., Chebaro Y. C., Knowles T. P. J. & Frenkel D. Crucial role of nonspecific interactions in amyloid nucleation. Proc. Natl. Acad. Sci. USA 111, 17869–17874 (2014). 25453085 10.1073/pnas.1410159111 PMC4273401

[b28] Nelson R. *et al.* Structure of the cross β spine of amyloid-like fibrils. Nature 435, 773–778 (2005). 15944695 10.1038/nature03680 PMC1479801

[b29] Bieler N. S., Knowles T. P. J., Frenkel D. & Vácha R. Connecting macroscopic observables and microscopic assembly events in amyloid formation using coarse grained simulations. PLoS Comput. Biol. 8, e1002692 (2012). 23071427 10.1371/journal.pcbi.1002692 PMC3469425

[b30] Ranjith P., Lacoste D., Mallick K. & Joanny J. F. Nonequilibrium self-assembly of a filament coupled to ATP/GTP hydrolysis. Biophys. J. 96, 2146–2159 (2009). 19289041 10.1016/j.bpj.2008.12.3920 PMC2717352

[b31] Gillespie D. T. Exact stochastic simulation of coupled chemical reactions. J. Phys. Chem. 81, 2340–2361 (1977).

[b32] Footer M. J., Kerssemakers J. W. J., Theriot J. A. & Dogterom M. Direct measurement of force generation by actin filament polymerization using an optical trap. Proc. Natl. Acad. Sci. USA. 104, 2181–2186 (2007). 17277076 10.1073/pnas.0607052104 PMC1892916

[b33] Mogilner A. & Oster G. Cell motility driven by actin polymerization. Biophys. J. 71, 3030–3045 (1996). 8968574 10.1016/S0006-3495(96)79496-1 PMC1233792

[b34] Inoué S. & Salmon E. D. Force Generation by Microtubule Assembly/Disassembly in Mitosis and Related Movements. Mol. Biol. Cell. 6, 1619–1640 (1995). 8590794 10.1091/mbc.6.12.1619 PMC301321

[b35] Dogterom M., Kerssemakers J. W. J., Romet-Lemonne G. & Janson M. E. Force generation by dynamic microtubules. Curr. Opin. Cell Biol. 17, 67–74 (2005). 15661521 10.1016/j.ceb.2004.12.011

[b36] Herling T. W. *et al.* Force generation by the growth of amyloid aggregates. Proc. Natl. Acad. Sci. USA 201417326 (2015). 10.1073/pnas.1417326112 PMC4534261 26195762

[b37] Dogterom M. & Yurke B. Measurement of the Force-Velocity Relation for Growing Microtubules. Science 278, 856–860 (1997). 9346483 10.1126/science.278.5339.856

[b38] Tanaka M. & Komi Y. Layers of structure and function in protein aggregation. Nat. Chem. Biol. 11, 373–377 (2015). 25978986 10.1038/nchembio.1818

[b39] Ahmed M. *et al.* Structural conversion of neurotoxic amyloid β(1–42) oligomers to fibrils. Nat. Struct. Mol. Biol. 17, 561–567 (2010). 20383142 10.1038/nsmb.1799 PMC2922021

[b40] Pellarin R., Guarnera E. & Caflisch A. Pathways and intermediates of amyloid fibril formation. J. Mol. Biol. 374, 917–924 (2007). 18028943 10.1016/j.jmb.2007.09.090

[b41] Fändrich M. Oligomeric Intermediates in Amyloid Formation: Structure Determination and Mechanisms of Toxicity. J. Mol. Biol. 421, 427–440 (2012). 22248587 10.1016/j.jmb.2012.01.006

[b42] Bernstein S. L. *et al.* Amyloid-*β* protein oligomerization and the importance of tetramers and dodecamers in the aetiology of Alzheimer’s disease. Nat. Chem. 1, 326–331 (2009). 20703363 10.1038/nchem.247 PMC2918915

[b43] Urbanc B., Betnel M., Cruz L., Bitan G. & Teplow D. B. Elucidation of amyloid *β*-protein oligomerization mechanisms: Discrete molecular dynamics study. J. Am. Chem. Soc. 132, 4266–4280 (2010). 20218566 10.1021/ja9096303 PMC5767167

[b44] Ono K., Condron M. M. & Teplow D. B. Structure–neurotoxicity relationships of amyloid *β*-protein oligomers. Proc. Natl. Acad. Sci. USA 106, 14745–14750 (2009). 19706468 10.1073/pnas.0905127106 PMC2736424

[b45] Arosio P., Beeg M., Nicoud L. & Morbidelli M. Time evolution of amyloid fibril length distribution described by a population balance model. Chem. Eng. Sci. 78, 21–32 (2012).

[b46] Nicoud L., Lazzari S., Barragán D. B. & Morbidelli M. Fragmentation of amyloid fibrils occurs in preferential positions depending on the environmental conditions. J. Phys. Chem. B 119, 4644–4652 (2015). 25792156 10.1021/acs.jpcb.5b01160

[b47] Ban T. *et al.* Direct observation of A β amyloid fibril growth and inhibition. J. Mol. Biol. 344, 757–767 (2004). 15533443 10.1016/j.jmb.2004.09.078

[b48] Nag S. *et al.* Nature of the amyloid β monomer and the monomer-oligomer equilibrium. J. Biol. Chem. 286, 13827–13833 (2011). 21349839 10.1074/jbc.M110.199885 PMC3077583

[b49] Jha N. N. *et al.* Characterization of amyloid formation by glucagon-like peptides: role of basic residues in heparin-mediated aggregation. Biochemistry 52, 8800–10 (2013). 24236650 10.1021/bi401398k

[b50] Baldwin A. J. *et al.* Metastability of Native Proteins and the Phenomenon of Amyloid Formation. J. Am. Chem. Soc. 133, 14160–14163 (2011). 21650202 10.1021/ja2017703

[b51] Dong J., Shokes J. E., Scott R. A. & Lynn D. G. Modulating Amyloid Self-Assembly and Fibril Morphology with Zn(II). J. Am. Chem. Soc. 128, 3540–3542 (2006). 16536526 10.1021/ja055973j PMC3555692

[b52] Anoop A. *et al.* Elucidating the role of disulfide bond on amyloid formation and fibril reversibility of somatostatin-14: Relevance to its storage and secretion. J. Biol. Chem. 289, 16884–16903 (2014). 24782311 10.1074/jbc.M114.548354 PMC4059132

[b53] Morel B., Varela L., Azuaga A. I. & Conejero-Lara F. Environmental conditions affect the kinetics of nucleation of amyloid fibrils and determine their morphology. Biophys. J. 99, 3801–3810 (2010). 21112305 10.1016/j.bpj.2010.10.039 PMC2998616

[b54] Singh P. K. *et al.* Cytotoxic Helix-Rich Oligomer Formation by Melittin and Pancreatic Polypeptide. Plos One 10, e0120346 (2015). 25803428 10.1371/journal.pone.0120346 PMC4372375

[b55] Bleiholder C., Dupuis N. F., Wyttenbach T. & Bowers M. T. Ion mobility–mass spectrometry reveals a conformational conversion from random assembly to *β*-sheet in amyloid fibril formation. Nat. Chem. 3, 172–177 (2011). 21258392 10.1038/nchem.945 PMC3073516

[b56] Schmit J. D., Ghosh K. & Dill K. What drives amyloid molecules to assemble into oligomers and fibrils? Biophys. J. 100, 450–8 (2011). 21244841 10.1016/j.bpj.2010.11.041 PMC3021675

[b57] Das A. K. *et al.* An early folding contact between phe19 and leu34 is critical for amyloid β oligomer toxicity. ACS Chem. Neurosci. 6, 1290–1295 (2015). 25951510 10.1021/acschemneuro.5b00074

[b58] Cheon M. *et al.* Structural reorganisation and potential toxicity of oligomeric species formed during the assembly of amyloid fibrils. PLoS Comput. Biol. 3, e173 (2007). 10.1371/journal.pcbi.0030173 PMC1976335 17941703

[b59] Cohen S. I. A. *et al.* Proliferation of amyloid-beta42 aggregates occurs through a secondary nucleation mechanism. Proc. Natl. Acad. Sci. USA 110, 9758–9763 (2013). 23703910 10.1073/pnas.1218402110 PMC3683769

[b60] Cohen S. I. A., Vendruscolo M., Dobson C. M. & Knowles T. P. J. From macroscopic measurements to microscopic mechanisms of protein aggregation. J. Mol. Biol. 421, 160–71 (2012). 22406275 10.1016/j.jmb.2012.02.031

