# Supplementary material for: A minimal conformational switching-dependent model for amyloid self-assembly
Source: Sci Rep. 2016 Feb 17;6:21103. doi: 10.1038/srep21103 (PMC4756677; doi:10.1038/srep21103)
Supplement: Supplementary Information [file srep21103-s1.pdf]

A minimal conformational switching-dependent model for amyloid self-assembly

Srivastav Ranganathan, Dhiman Ghosh, Samir K Maji, Ranjith Padinhateeri

Supplementary Text S1

Analytical solution for filament and β-sheet growth velocities

$$\frac{dP_0}{dt} = P_1(k_{cb}^- + k_{cb}^s) - P_0(k_{cb}^+ C_s + k_{bc}^s) \tag{1}$$

$$\frac{dP_1}{dt} = P_0(k_{cb}^+ C_s + k_{bc}^s) + P_2(k_{cc}^- + k_{cb}^s) - (k_{cc}^+ C_s + k_{cb}^s + k_{bc}^s + k_{cb}^-)P_1 \tag{2}$$

for $k > 1$,

$$\frac{dP_k}{dt} = P_{k-1}(k_{cc}^+ C_s + k_{bc}^s) + P_{k+1}(k_{cc}^- + k_{cb}^s) - P_k(k_{cc}^+ C_s + k_{cc}^- + k_{bc}^s + k_{cb}^s) \tag{3}$$

$$P_0 + P_1 + P_2 + P_3 + ..P_\infty = 1 \tag{4}$$

$$P_0 + P_1 + A\sum_{k=2}^{n} q^k = 1 \tag{5}$$

$$A = \frac{1 - P_0 - P_1}{\sum_{k=2}^{n} q^k} \tag{6}$$

where,

$$\sum_{k=2}^{n} q^k = \frac{q^2}{1-q} \tag{7}$$

Note that, the above equation is valid only if $k_{cc}^- + k_{cb}^s >= k_{cc}^+ C_s$.
We set $k_{bc}^s = 0$ in order to neglect the contribution of backward switching from state B to state C.

$$q = \frac{k_{cc}^+ C_s}{k_{cc}^- + k_{cb}^s} \tag{8}$$

Now, at steady state,

$$\frac{dP_0}{dt} = \frac{dP_1}{dt} = 0 \tag{9}$$

$$P_1 = P_0 \frac{k_{cb}^+ C_s}{k_{cb}^- + k_{cb}^s} \tag{10}$$

Solving for P_0 and P_1 we get,

$$P_0 = \frac{(k_{cb}^- k_{cc}^- + k_{cb}^- k_{cb}^s + k_{cc}^- k_{cb}^s + (k_{cb}^s)^2 - k_{cb}^- k_{cc}^+ C_s - C_s k_{cc}^+ k_{cb}^s)}{(k_{cb}^- k_{cc}^- + k_{cb}^- k_{cb}^s + k_{cc}^- k_{cb}^s + (k_{cb}^s)^2 - k_{cb}^- k_{cc}^+ C_s + k_{cc}^- k_{cb}^+ C_s + k_{cb}^+ k_{cb}^s C_s - k_{cc}^+ k_{cb}^s C_s)} \tag{11}$$

$$P_1 = P_0 \frac{k_{cb}^+ C_s}{k_{cb}^- + k_{cb}^s} \tag{12}$$

Expressions for velocities in the two regimes:

Regime 1 (for $q < 1$): The filament growth velocities in the first regime ($q < 1$), under the steady state assumption ($dP_k/dt = 0$) is given by:

$$v_f = P_0 k_{cb}^+ C_s + P_1 (k_{cc}^+ C_s - k_{cb}^-) + (1 - P_0 - P_1)(k_{cc}^+ C_s - k_{cc}^-) \tag{13}$$

and

$$v_b = (1 - P_0) k_{cb}^s \tag{14}$$

Regime 2 (q = 1 and $k_{cc}^+ C_s \geq k_{cc}^- + k_{cb}^s$)

In the second regime (q=1), $dP_k/dt \neq 0$. Therefore, we cannot use the earlier steady state assumption that was valid for regime 1. The filament growth velocities in this regime can be derived without any steady-state assumption. In this regime, the filament growth depends only on the growth of the 'C' monomers. Therefore, one can write a master equation for G_k which is the probablity of finding a filament with total length k:

$$dG_k/dt = k_{cc}^+ C_s G_{k-1} + k_{cc}^- G_{k+1} + (k_{cc}^+ C_s + k_{cc}^-) G_k \tag{15}$$

The above expression can be solved by performing a transformation (also see reference [1] below) given by:

$$F(\lambda, t) = \sum_k G_k(t) e^{-k\lambda} \tag{16}$$

Doing this kind of a z-transformation, we obtain,

$$dF(\lambda, t)/dt = [k_{cc}^+ C_s (e^{-\lambda} - 1) + k_{cc}^- (e^{-\lambda} - 1)] F(\lambda, t). \tag{17}$$

The above expression can be solved under to obtain a relationship for the velocity such that,

$$v_f = \frac{d}{dt} \left(\frac{\partial F}{\partial \lambda} \right)_{\lambda \to 0} = k_{cc}^+ C_s - k_{cc}^- \tag{18}$$

$$v_f = k_{cc}^+ C_s - k_{cc}^- \tag{19}$$

References

[1] Padinhateeri Ranjith, David Lacoste, Kirone Mallick and Jean François Joanny. *Nonequilibrium self-assembly of a filament coupled to ATP/GTP hydrolysis.* Biophys. J . **96** (6), 2146–2159 (2009).

Supplementary Text S2

Computing the force-velocity relationship

In order to compute the effect of external force on the growth velocity, we use modified forms of the velocity equations to account for the effect of the external force on the polymerization and depolymerization rates. Let $v_f(F)$ be the growth velocity in the presence of an external force, 'F'. The modified form of the velocity equation are shown in expressions 20 and 21.

$$v_f(F) = P_0 k_{cb}^{+}(F) C_s + P_1(k_{cc}^{+}(F) C_s - k_{cb}^{-}(F)) + (1 - P_0 - P_1)(k_{cc}^{+}(F) C_s - k_{cc}^{-}(F)) \tag{20}$$

and

$$v_f(F) = k_{cc}^{+}(F) C_s - k_{cc}^{-}(F) \tag{21}$$

Here, $k_{cc}^{+}(F)$,$k_{cc}^{-}(F)$,$k_{cb}^{+}(F)$ and $k_{cb}^{-}(F)$ are rescaled rate constants in the response to external force, and the expressions for the same are given in equations 10-13 in the main text.

Model setup for the fibril breakage simulations

Two-state model with fibril breakage: In addition to the aforementioned model with events like polymerization, depolymerization and conformational switching, we also performed simulations wherein any growing filament could also undergo breakage with certain rate, k_{break}. Upon breaking, the two resultant filaments would both continue to grow as per the our model setup described before. The location of the break in our simulations was close to the center of the fibril. The setup of the fibril breakage simulations is schematically depicted in Supplementary Figure S9.A. simulations involving fibril breakage were performed under the constant free monomer concentration conditions. These simulations were performed to emphasize the fact that under these conditions of constant free monomer concentration, any resulting seed formed as a result of fibril breakage would continue to grow at velocities that can be predicted by our analytical theory.

Fitting the velocity-concentration curve to experiment

In order to fit the concentration-velocity profile to experiment (Figure. 3 of the main text), the following rationale was used. Firstly, not all 5 parameters are mandatory to obtain a good fit. However, a certain set of relations between the magnitude of rate constants must be satisfied in order to obtain the fit. More importantly, irrespective of the value of other parameters, the saturation value of the velocity is dictated by just one parameter—the rate of conformational switching. Therefore, we can equate the saturation velocity to the switching rate and obtain k_{cb}^{s} from the saturation values. Since, the saturation of growth velocities is a phenomenon that emerges in the first regime of growth ($C_s < [C]*$), the ratio of magnitudes of rates is such that, $k_{cc}^{+}/k_{cc}^{-} << k_{cb}^{+}/k_{cb}^{-}$. Upon satisfying this condition, a large range of values could be used to fit the curve to the experiment. However, the ratio of magnitudes of the rates, k_{cb}^{+}/k_{cb}^{-} must be a constant value.

The emphasis of the plot (Figure. 3 of the main text) is therefore to ascertain the switching rate, which dictates the maximum attainable velocity in the first regime. In order to obtain the absolute values of model parameters like $k_c c^{+}$ and $k_c c^{-}$, one would need to perform experiments spanning both the growth regimes ($C_s < [C]*$, and $C_s > [C]*$).

Supplementary Text S3

Experimental Methods

Amyloid fibril formation

The aggregation kinetics was initiated with LMW synuclein at a concentration of 300 M in 1.5 ml eppendorf tube in 20 mM Gly-NaOH, pH 7.4, 0.01% sodium azide. The eppendorf tubes containing protein solutions were placed into an Echo Therm model RT11 rotating mixture (Torrey Pines Scientific, USA) with a speed corresponding to 50 r.p.m. inside a 37 degrees C incubator.

ThT fluorescence assay

1 mM ThT was prepared in Tris-HCl buffer, pH 8.0, 0.01% sodium azide. 3.3 μl of the protein solution was taken and diluted to 200 μl Gly-NaOH buffer, pH 7.4, 0.01% sodium azide such that final protein concentration becomes 5 μM. 2 μl of 1 mM ThT solution was added to the 5 μM protein solution in 200 μl Gly-NaOH buffer, pH 7.4, 0.01% sodium azide. Immediately after addition, ThT fluorescence assay was measured using Horiba-Jobin Yvon (Fluomax4) with excitation at 450 nm and emission in the range of 460-500 nm. The slit width for both excitation and emission were kept at 5 nm. ThT fluorescence obtained at 480 nm was plotted against incubation time and the data were fitted to a sigmoidal curve.

Atomic force microscopy (AFM)

Time dependent morphological changes during synuclein aggregation kinetics was performed using AFM (Asylum research, USA). At three different time points (i.e 0h, beginning of lag phase; 96h, middle of elongation phase and 180h, saturation phase) during aggregation, a small aliquot of incubated samples were taken out, diluted to a final concentration of 30 μM and spotted on a freshly cleaved mica sheet and incubated at room temperature for 5 minutes. Unbound proteins/aggregates were washed by distilled water and dried under vacuum. The imaging was performed in tapping mode with silicon cantilever with a scan rate 1 Hz and around 6 different areas were scanned randomly.

Supplementary Figures

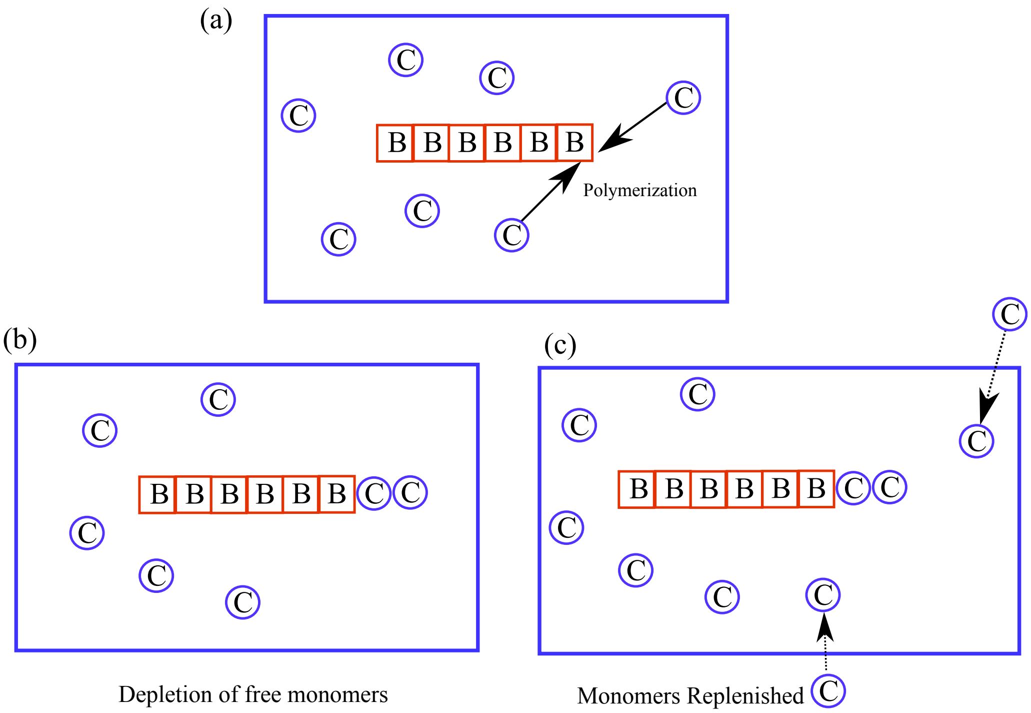

Figure S1. Simulation setups. A) Initial state of the system with monomers polymerizing onto a growing fibril. B) Depletion of free monomers upon polymerization in the 'Mass Conserved' setup and C) the 'Constant Concentration' setup wherein C monomers are replenished once they polymerize onto the growing filament, thereby always maintaining a constant number of free monomers throughout the simulation.

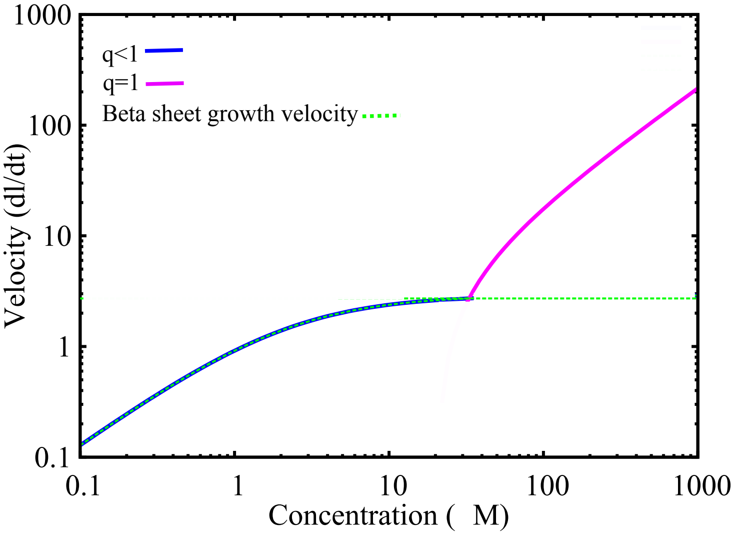

Figure S2. Comparison of beta-sheet and overall aggregate growth velocities. In the first regime, the filament extension is mainly the growth of the beta sheet which is evident by the overlapping curves (solid blue and dotted green). However, in the second regime the aggregate grows mainly by polymerizing C monomers while the rate of β-sheet development is constant and equal to the switching rate, k^s_{cb}.

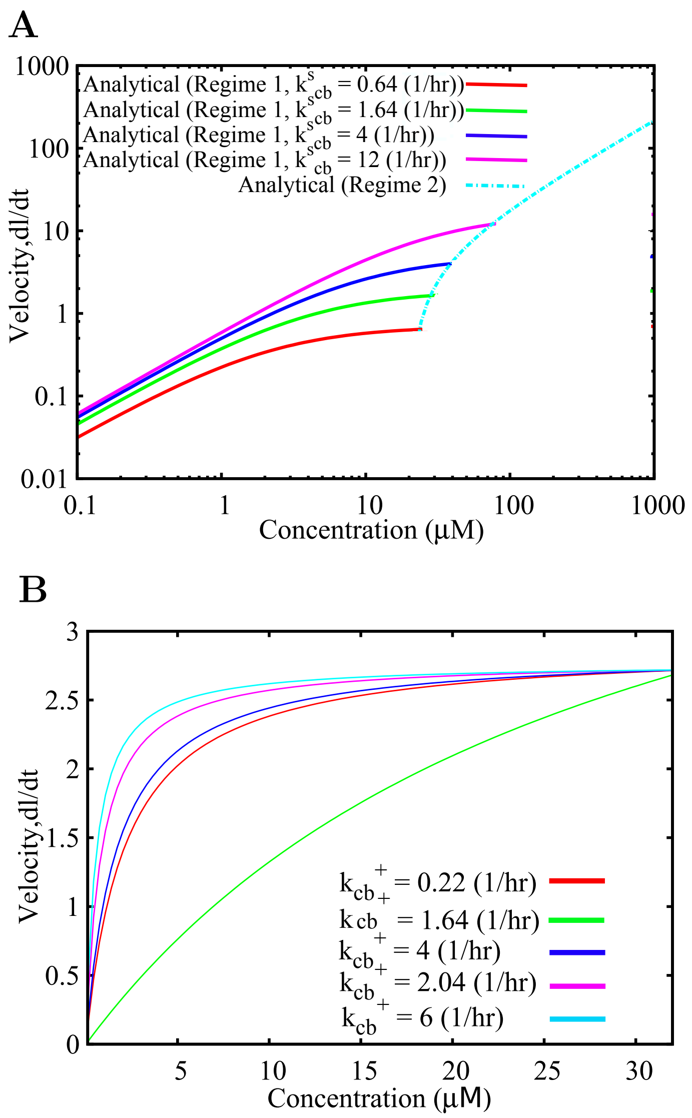

Figure S3. Effect of parameters on concentration-velocity profile. Effect of A) varying switching rate and B) varying k_{cb}^{+} on the concentration-velocity curve.

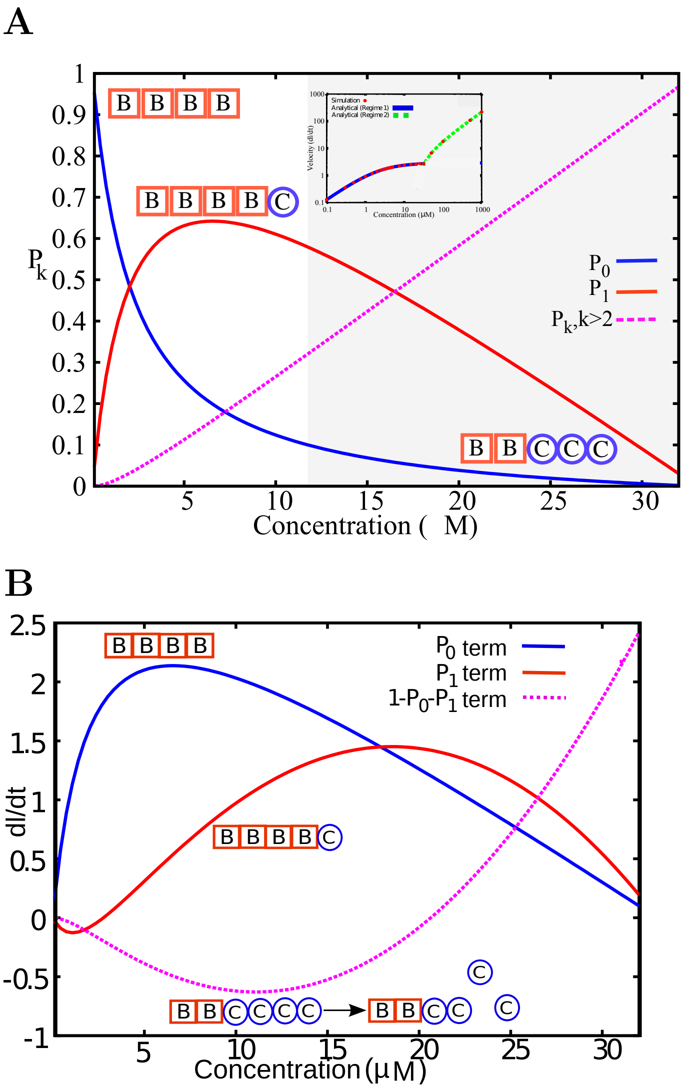

Figure S4. Behavior of probabilities and individual terms in the velocity expression in response to concentration. A) The probability of finding 0, 1 and more than one 'C' monomers at the tip of a growing filament and the effect of concentration on the probabilities. At lower concentrations, there is a high probability of encountering filaments which are entirely in state B. At higher concentrations, P_1 and $1 - P_0 - P_1$ start dominating over P_0, and B) Behavior of the P_0,P_1 and $1 - P_0 - P_1$ terms in the velocity expression with increasing free monomer concentration, C_s.

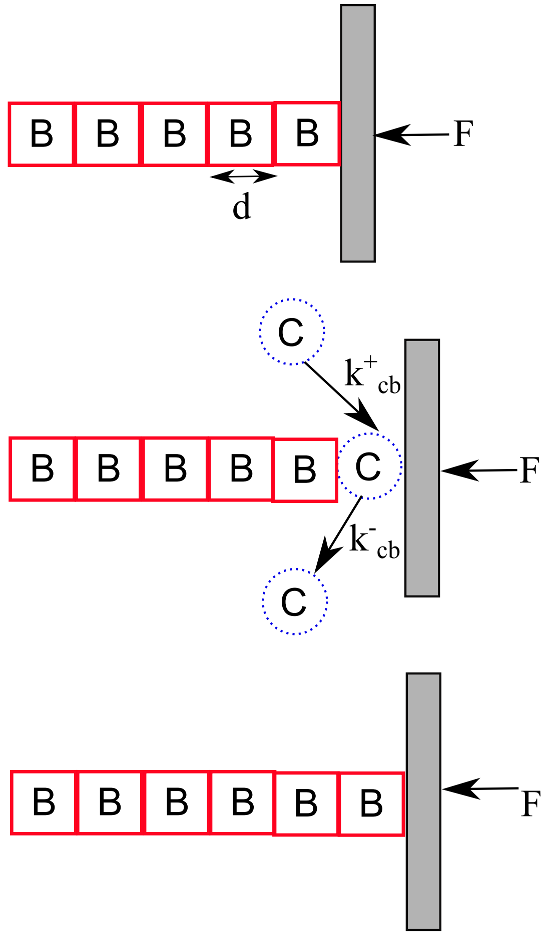

Figure S5. Filament growth against external force. Schematic representation of the Brownian rachet principle for force-generating polymerizing amyloid fibrils. The filament growth occurs against an external force 'F' applied by a wall which is constantly undergoing thermal fluctuations. The fibril can grow as long as the space between the tip of the fibril and the wall is greater than the size of a subunit 'd'. The rates of polymerization and depolymerization are k^+_{cb} and k^-_{cb} respectively. Thermal fluctuation of the wall could occassionally allow for the extension of the fibril by addition of monomers.

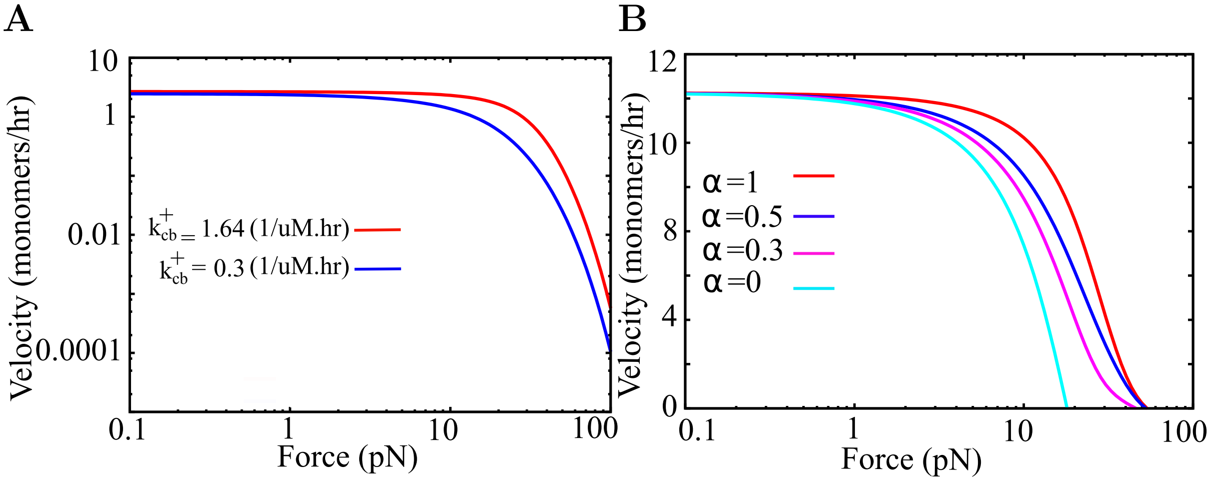

Figure S6. The force-velocity relationship. A) For lower k_{cb}^{+} values, we observe a sharper decline in the growth velocities in response to external force (blue curve). B) For different values of α, we observe variations in the stall forces. Any variation in α would result in differences in the manner in which the force gets distributed between the polymerization and depolymerization events thereby resulting the different profiles.

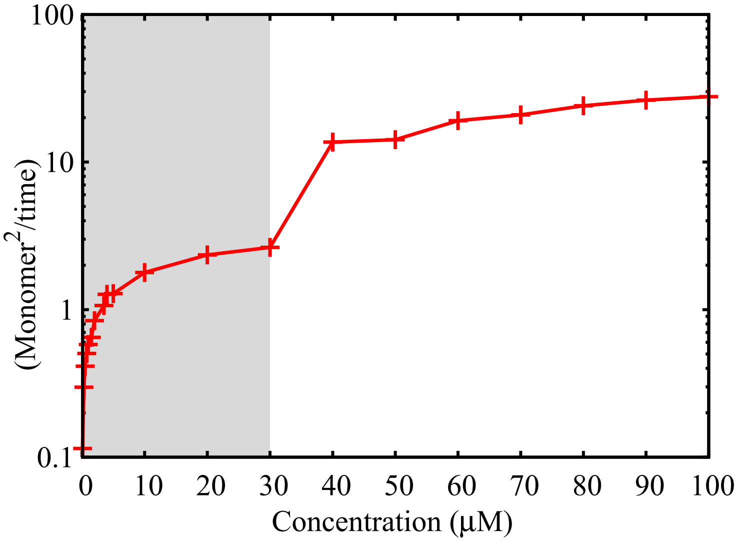

Figure S7. Diffusion coefficients for various free monomer concentration. Diffusion coefficients indicating the extent of heterogenity in aggregate length as a function of concentration. The first regime (shaded region) of switching dependant growth shows a saturation in length variation which later increases linearly as concentration increases beyond the critical concentration.

A

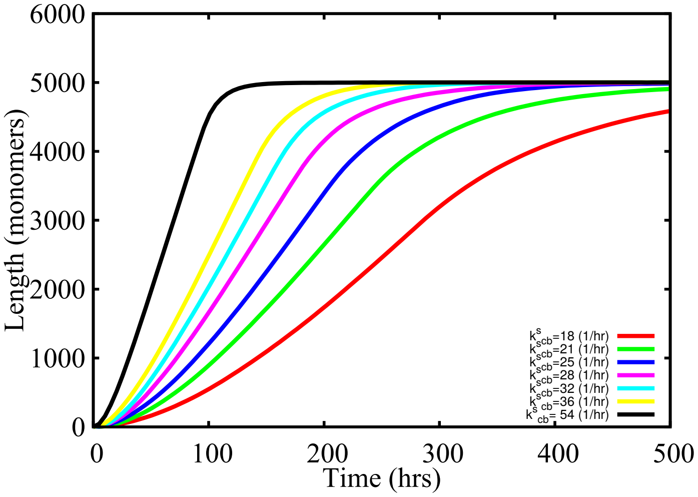

B

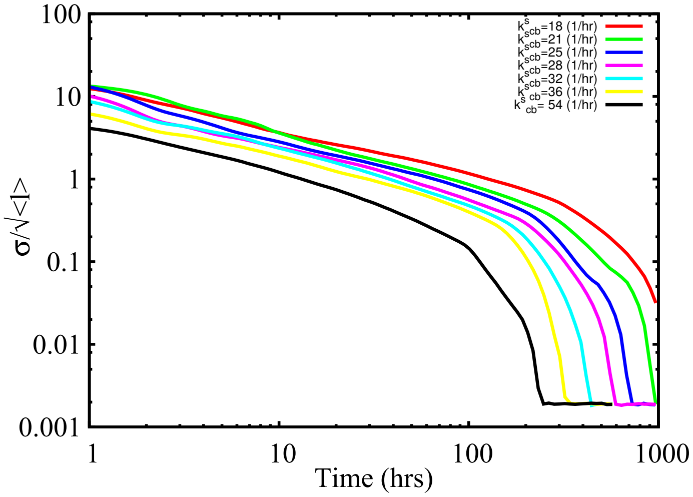

Figure S8. Mass conserved growth kinetics. A) Effect of switching rate on the growth kinetics of the fibrils under the mass conserved setup and B) time progression of fibril length heterogenity, characterized by $\sigma / < l >$ plotted as a function of time.

A

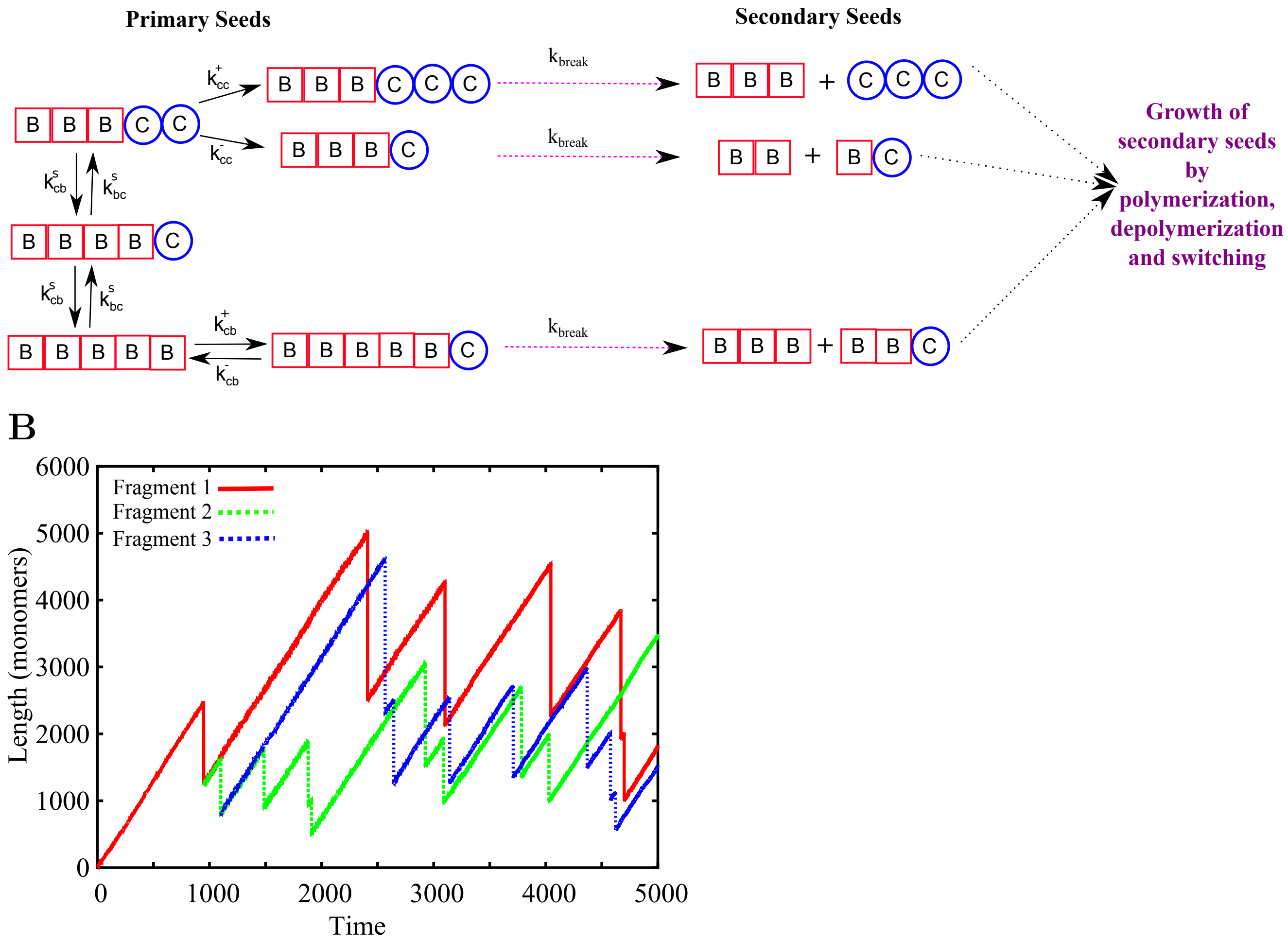

Figure S9. Growth profiles for fragments upon introducing fibril breakage. Length profiles for filaments under constant concentration condition upon introduction of fibril breaking rate in the model. Each new filament grows with the same steady state velocity as evident from their identical slopes under the constant concentration condition.
